# Posterior Reversible Encephalopathy Syndrome due to High Dose Corticosteroids for an MS Relapse

**DOI:** 10.1155/2015/325657

**Published:** 2015-05-26

**Authors:** Sarah A. Morrow, Robina Rana, Donald Lee, Terri Paul, Jeffrey L. Mahon

**Affiliations:** ^1^Department of Clinical Neurological Sciences, University of Western Ontario, 339 Windermere Road, London, ON, Canada N6A 5A5; ^2^Department of Medicine, University of Western Ontario, 339 Windermere Road, London, ON, Canada N6A 5A5

## Abstract

Increased blood pressure is a known adverse effect associated with corticosteroids but little is published regarding the risk with the high doses used in multiple sclerosis (MS). A 53-year-old female with known relapsing remitting MS presented with a new brainstem relapse. Standard of care treatment for an acute MS relapse, 1250 mg of oral prednisone for 5 days, was initiated. She developed an occipital headache and dizziness and felt generally unwell. These symptoms persisted after treatment was complete. On presentation to medical attention, her blood pressure was 199/110 mmHg, although she had no history of hypertension. MRI changes were consistent with posterior reversible encephalopathy syndrome (PRES), demonstrating abnormal T2 signal in both thalami, the posterior occipital and posterior parietal white matter with mild sulcal effacement. As her pressure normalized with medication, her symptoms resolved and the MRI changes improved. No secondary cause of hypertension was found. This is the first reported case of PRES secondary to high dose corticosteroid use for an MS relapse without a history of hypertension and with no other secondary cause of hypertension identified. This rare complication should be considered in MS patients presenting with a headache or other neurological symptoms during treatment for a relapse.

## 1. Introduction

Posterior reversible encephalopathy syndrome (PRES) is a sporadic disease most commonly associated with an acute increase in blood pressure [1]. Presenting symptoms include headache, visual changes, vomiting, seizure, and confusion [2]. There are many medical conditions and medications that are known to increase the risk of acute hypertension, including corticosteroids (CR). We report a case of PRES due to high dose CR in an MS patient with an acute demyelinating event.

## 2. Case

A 53-year-old right handed female was diagnosed with relapsing remitting MS in 2011 after clinical relapses in 2006, 2010, and 2011 and an MRI consistent with demyelination, none of which were treated with CR. She was started on interferon *β*-1a 30 *μ*g IM weekly, a disease modifying therapy for MS, shortly after diagnosis. She had a sensory relapse in 2012 that resolved without CR treatment. Past medical history was significant for chronic calcific pancreatitis that was not autoimmune in nature; investigations by gastroenterology were negative and it was felt to be idiopathic in nature. There was no history of alcohol abuse or acute pancreatitis, despite a past history of a cholecystectomy. There was no known history of hypertension (HTN) and her blood pressure (BP) in the past was around 125/85 mmHg.

**Figure 1:**
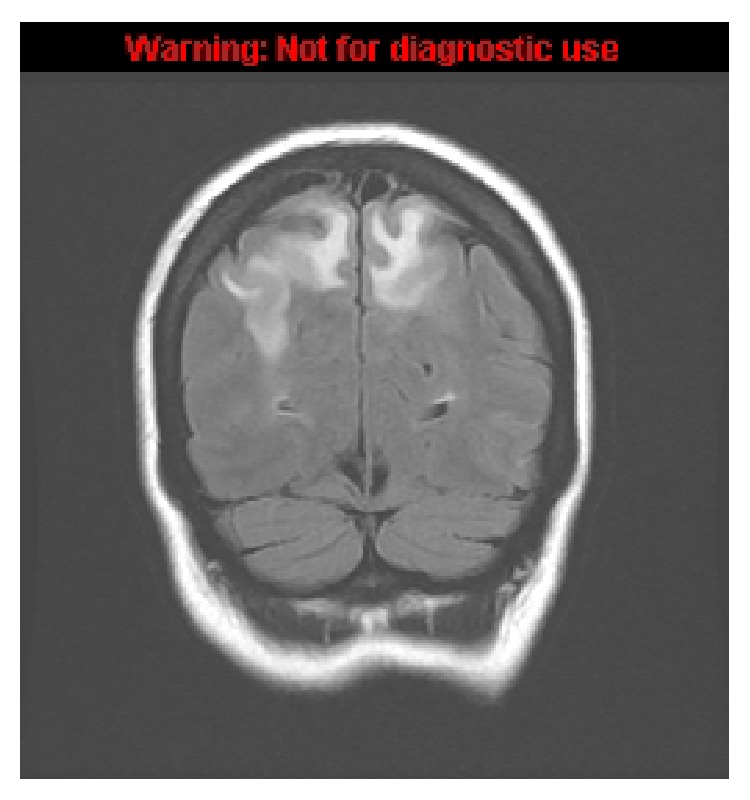
Coronal FLAIR image demonstrating hyperintensity in the posterior occipital white matter extending up to the vertex with mild local sulcal effacement consistent with posterior reversible encephalopathy syndrome (PRES).

In June 2013 she presented with diplopia. A new intranuclear ophthalmoplegia (INO) was identified and she was diagnosed with a relapse. Treatment with high dose CR was offered and she was started on 1250 mg oral prednisone for 5 days with no taper. On day one of CR, she noted insomnia, dizziness, general malaise, and a headache described as dull and gradual in onset. Upon completion of the pulse CR treatment, her symptoms persisted and she therefore came to the Emergency Department. She was not seen by a physician as she felt that the wait was too long and left. Over the following week, her headache worsened and she re-presented to the Emergency Department, describing the headache as starting in the occipital region and migrating over time to be holocephalic. The pain was rated as 10/10 but continued to be dull and aching. No nausea or vomiting, visual changes, or new neurologic symptoms were present. Her BP was 199/110 mmHg and heart rate was 78 beats per minute and regular and general medical examination was within normal limits. The neurologic examination was consistent with the findings prior to the onset of the relapse; her INO and diplopia had resolved. Investigations in the Emergency Department included a normal electrocardiogram with normal sinus rhythm and no evidence of left ventricular hypertrophy, normal urinalysis including no protein or blood, and unremarkable pelvic/renal ultrasound including no renal calculi. An MRI of the head and C-spine was performed. In addition to T2 hyperintensities in the brain and the cervical cord consistent with MS, unchanged since the previous MRI six months earlier, there was also abnormal T2 signal in both thalami, the posterior occipital and posterior parietal white matter extending into the vertex. Mild local sulcal effacement associated with these new lesions was also noted. There was no diffusion restriction noted with these lesions. The findings described above were consistent with a diagnosis of PRES (Figure 1). She was admitted to hospital; her BP was monitored and remained relatively unchanged for the next 48 hours despite treatment with hydrochlorothiazide and amlodipine. Her BP subsequently responded to labetalol and amlodipine and was stabilized at approximately 130/85 mmHg and her headache resolved. Investigations for other causes of PRES as well as secondary causes of HTN were performed. Laboratory investigations included serum sodium 135 mmol/L, serum potassium 3.0 mmol/L, serum creatinine 79 *μ*mol/L, serum urea 4.1 mmol/L, serum calcium 2.21 mmol/L, and serum albumin level 43 g/L. Her body mass index was 24 kg/m^2^, she did not have signs of Cushing's syndrome or thyroid gland disease, there were no changes of hypertensive retinopathy, and kidney function was normal. Anti-nuclear antibody (ANA) was negative. A urinalysis was negative for protein and hematuria. She was not pregnant. Her medications at presentation included nortriptyline and interferon B-1a only, and she was on no over-the-counter medications that affect BP or are associated with PRES. A 24-hour urine collection for free cortisol and fractionated metanephrines level was done in hospital 19 days after her first dose of CR and showed increases in the cortisol (942 nmol/d) and normetanephrine (666 *μ*mol/mol creatinine) levels but a normal metanephrine level (78 *μ*mol/mol creatinine). Three subsequent 24-hour urine collections for fractionated metanephrines, fractionated catecholamines, and dopamine levels, including one collected 2 weeks after stopping nortriptyline, were normal, ruling out nortriptyline as a potential factor in her increased BP or PRES. A second 24-hour urine free cortisol 113 days after her first dose of CR was normal (285 nmol/d). Thus, no secondary causes of HTN or PRES were found.

**Figure 2:**
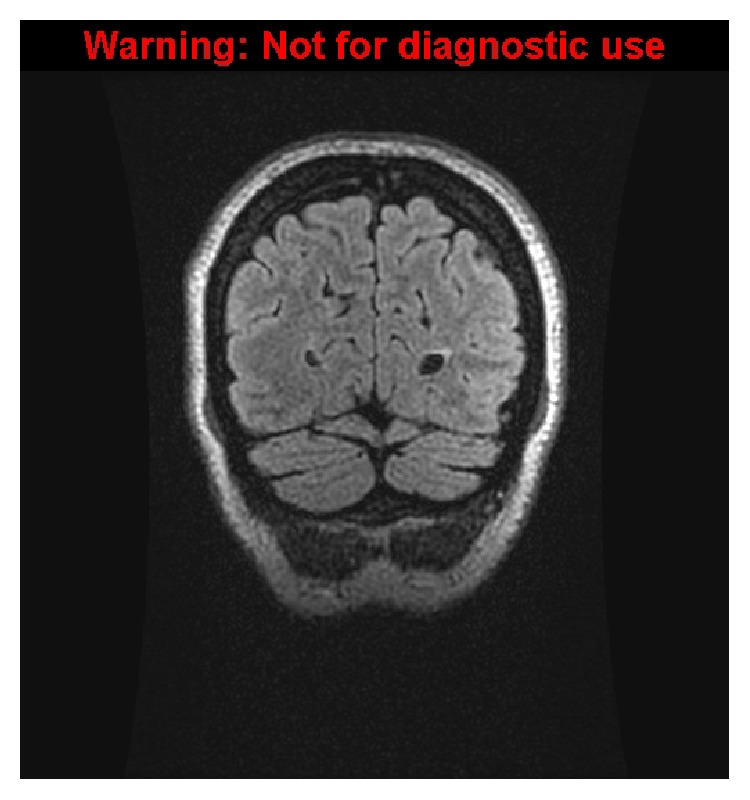
Follow-up coronal FLAIR image one month later demonstrating complete resolution of the hyperintensity noted previously.

Follow-up MRI one month later demonstrated resolution of the signal change in the posterior cerebral hemispheres, consistent with resolution of PRES (Figure 2). She was discharged on amlodipine 10 mg daily and labetalol 150 mg twice a day. She continued to be neurologically stable at her follow-up visit in the MS clinic one month after discharge with a blood pressure of 140/87 mmHg.

## 3. Discussion

PRES was first described by Hinchey et al. in 1996 but its true incidence is unknown [3]. Although the presenting symptoms can be severe, including visual loss, headaches, confusion, decreased level of consciousness, and/or seizures, and the syndrome is often completely reversible, there are some cases where full recovery has not occurred [2]. It is mainly associated with acute hypertension and with certain medical conditions such as electrolyte imbalance, vasoactive drugs, eclampsia/preeclampsia, sepsis, Guillain-Barre syndrome, autoimmune diseases and chemotherapy agents, and other immunosuppressive drugs [2, 4].

MRI is considered the gold standard for confirmation of diagnosis and typically demonstrates hyperintense T2 weighted and FLAIR lesions due to high water mobility, which appear normal on diffusion-weighted imaging predominantly occurring at the occipital regions [2]. Greater than 90% of patients demonstrate involvement of the parietooccipital lobes, but lesion can also be present in the frontal lobe basal ganglia, cerebellum, and brainstem in 1/3 of cases [2]. PRES lesions tend to be symmetric, affecting both hemispheres. The PRES lesions are thought to represent vasogenic edema, although the pathophysiology that leads to PRES is not known; one popular theory is that acute hypertension is associated with failed autoregulation, enhanced vascular permeability, and hyperperfusion which leads to edema [3, 5]. The mechanism underlying how hypertension can lead to PRES is not fully understood. It is postulated that acute hypertension is thought to exceed the upper limit of autoregulation, leading to vasodilation allowing leakage to occur leading to cerebral edema [6, 7].

The standard of care for acute multiple sclerosis (MS) relapses is high dose CR [8]. HTN is a known risk with the use of CR [9], possibly due to the effect of CR on the mineralocorticoid receptor [7]. There are several case reports of PRES being related to CR use. In most cases, however, the patient was also on another medication, such as CHOP or cisplatin chemotherapy for an underlying hematologic cancer [10, 11], or was acutely ill and normotensive [12] and thus a causal relationship between CR and PRES cannot be definitively made. There are two pediatric cases of PRES associated with pulses of high CR similar to our case, but in these cases, the patients were also acutely ill [13, 14]. In a prospective study of MS patients treated with 1 gram of methylprednisolone daily for 3–5 days, only 4/64 developed HTN [15]. To our knowledge, this is the first case of PRES reported in an MS patient secondary to high dose CR treatment for an acute relapse without any other potential etiologies present. There have been cases of PRES associated with neuromyelitis optica spectrum disorders, but none in the context of CR use. The authors of that case series postulated that interference with the aquaporin channel by the aquaporin-4 specific IgG may have contributed to the development of PRES [16].

It is well known that CR can cause HTN; a meta-analysis by Fardet et al. demonstrated an OR of 2.2 (95% CI 1.4–3.8) for developing HTN in patients treated with CR compared to controls [17]. However, this meta-analysis included studies with low dose CR (5–40 mg) over a long period of time (months to years). The exception was one study by Chibane et al. where patients were treated with high dose pulse CR, 500–1000 mg methylprednisolone, similar to the dose used for MS relapses, for various eye diseases [18]. The authors reported HTN, defined as BP ≥ 180/110 mmHg, in only 3.4% of subjects. Whether these patients were symptomatic was not specified. None of the above studies noted cases of PRES associated with this increase in BP due to CR.

This rare complication should be considered in MS patients presenting with a headache or other neurological symptoms during CR treatment.
